# Supplementary material for: Pharmacological Prevention of Ectopic Erythrophagocytosis by Cilostazol Mitigates Ferroptosis in NASH
Source: Int J Mol Sci. 2023 Aug 16;24(16):12862. doi: 10.3390/ijms241612862 (PMC10454295; doi:10.3390/ijms241612862)
Supplement: Supplementary file 1 [file ijms-24-12862-s001.zip › ijms-2536507-supplementary.pdf]

Table of Contents

Figure S1 3

Figure S2 4

SUPPLEMENTARY FIGURES

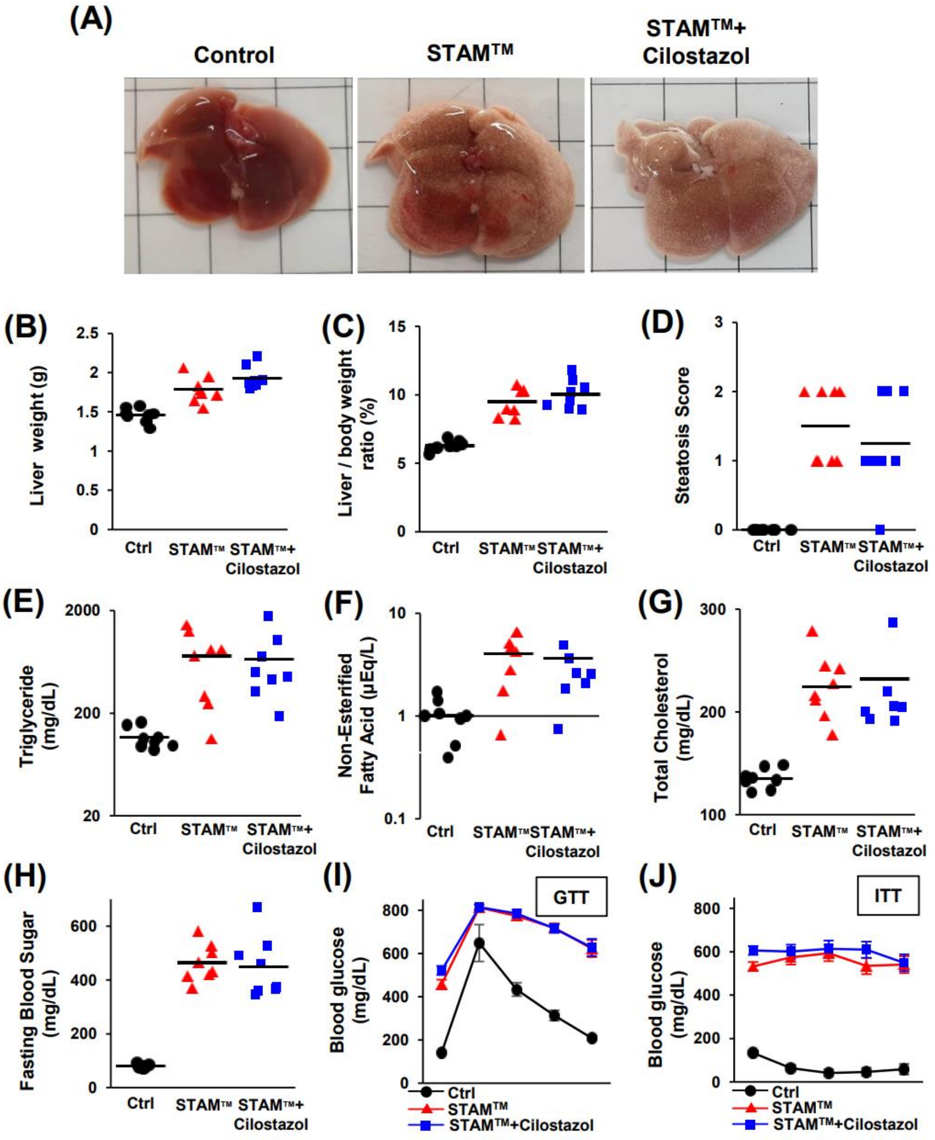

Figure S1. Cilostazol does not improve lipid and glucose profile in NASH mice. (**A**) Gross phenotype of liver. (**B**) Liver weight. (**C**) Liver weight / body weight. (**D**) steatosis score. (**E**) Triglyceride. (**F**) Non-esterified fatty acid. (**G**) Total cholesterol. (**H**) Fasting blood sugar. (**I**) Glucose tolerance test. (**J**) Insulin tolerance test.

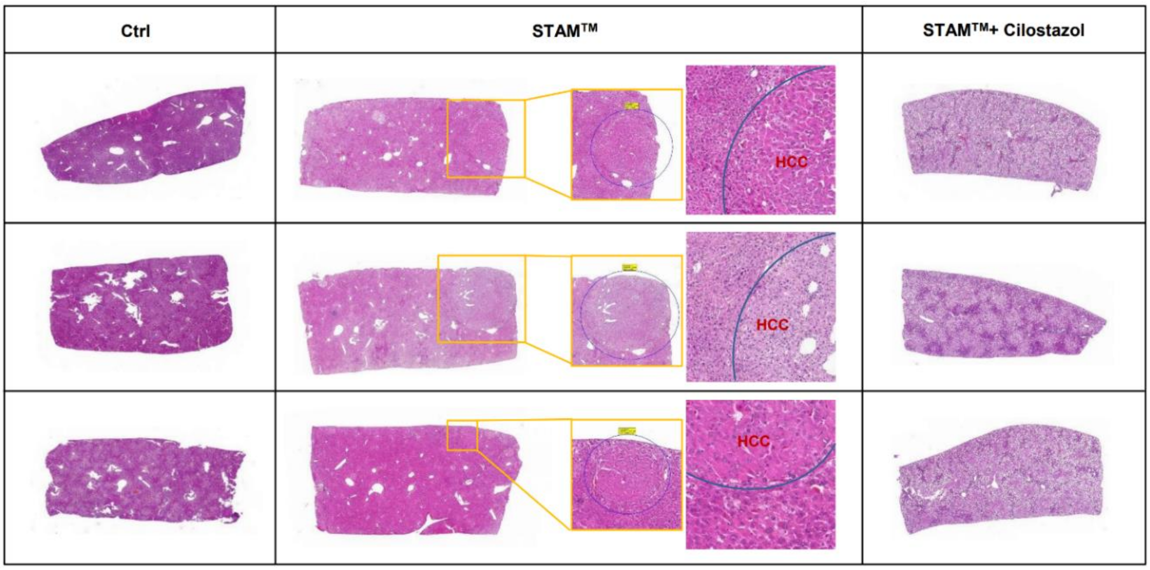

Figure S2. The effect of cilostazol on the progression of NASH to HCC in NASH mice. We performed the histological evaluation of liver tissue to investigate the role of cilostazol in NASH-induced HCC. The H&E slides of 14-week-old STAM^{TM} mice showed a well-circumscribed mass composed of cells with nuclear atypia forming the trabecular pattern, histologically consistent with well-differentiated HCC. In contrast, the sections of cilostazol-administered mice exhibited no definite mass-like lesion.
